# Enhancing canine semen quality through a second centrifugation after 48 hours of storage: a comparative study

**DOI:** 10.1186/s13028-024-00767-5

**Published:** 2024-09-11

**Authors:** Letizia Sinagra, Angela Polisca, Giulia Donato, Tiziana Caspanello, Giorgia Pettina, Sara Pastore, Massimo De Majo, Santo Cristarella, Marco Quartuccio, Viola Zappone

**Affiliations:** 1 Department of Veterinary Sciences, University of Messina, Viale Palatucci, 13, Messina, 98168 Italy; 2 Department of Veterinary Medicine, University of Perugia, Via San Costanzo 4, Perugia, 06126 Italy

**Keywords:** Artificial insemination, Cooled sperm, Dog, Sperm improving, Sperm motility

## Abstract

**Background:**

Centrifugation is a common procedure to improve the quality of chilled and frozen canine semen by removing debris and seminal plasma and adding semen extenders. The aim of this study was to evaluate the efficacy and influence of a second centrifugation after 48 h of storage at 5 °C on the sperm quality of canine semen. The ejaculates of 45 healthy male dogs, divided into three groups according to body weight, were analyzed for macro- and microparameters such as ejaculate volume, sperm concentration, kinematic parameters, morphology, and integrity of plasma membrane. Samples were analyzed at baseline conditions (T_0_), after 24 h (T_24_) and after 48 h (T_48_) to assess the effects of the different treatments on sperm quality.

**Results:**

The results showed a significant effect of a second centrifugation on the improvement of chilled sperm quality compared to the other techniques, especially up to 48 h.

**Conclusions:**

Analysis of the data showed that the semen samples centrifuged and then cooled at 5 °C had acceptable semen parameters, especially in terms of motility, with a gradual decrease in serial evaluations after 24 and 48 h. A second centrifugation after 48 h of storage may lead to better semen quality and improve the kinetics of sperm parameters, the percentage of morphologically normal sperm and the percentage of sperm with intact membranes.

## Background

Sperm evaluation includes both macro- and microanalysis, examining various factors such as volume, colour, pH, concentration, total sperm count (TSC), total sperm motility (TSM) and progressive sperm motility (PSM), as well as additional tests such as viability (using live-dead staining or the hypo-osmotic swelling test), morphological abnormalities, evaluation of other cell types and DNA analysis [1]. This evaluation technique is widely used to assess male fertility in both humans and animals [2–5].

With the increased use of artificial insemination (AI) techniques, the importance of sperm quality has grown, especially for cooled or frozen sperm. Indeed, better semen quality is associated with better fertility, in addition to optimal management factors and veterinary care [6]. The success of artificial insemination depends on the presence of a sufficient number of fertile sperm in the female genital tract [7]. To achieve favourable results, a minimum motility of 50% is required for fresh sperm in AI [8]. If only low-quality sperm is available, the increase in sperm concentration per dose of AI may result in litters comparable in size to those of females inseminated with high-quality semen [9]. One of the major advantages of cooled or frozen semen is the possibility to transport only the semen intended for AI to the recipient bitch, rather than the donor [10, 11]. Compared to freezing, refrigeration also offers a simpler cooling technique and cheaper transport [12]. However, sperm survival is reduced with chilling, compared to fresh semen [13]. On the other hand, sperm metabolism is highest at body temperature and starts to decrease at room temperature (24–29 °C); then it decreases to 50% every 10 °C and reaches the 10% of metabolism at 5 °C [14]. For this reason, in case of chilling and freezing semen, the addition of extenders is necessary to provide energy, maintain pH and osmolarity, and protect the acrosome and plasma membrane integrity against damage [10]. This can ensure the maintenance of the fertilizing ability of the sperm for a short period of time and achieve a success rate of 83.8% in artificial insemination [15].

The biochemical composition of seminal plasma is highly variable. There are controversial opinions in the literature regarding the preservation of seminal plasma together with spermatozoa [16]. In a study conducted in dogs it was reported that the percentage of morphologically intact spermatozoa after 6 h of incubation at 37 ºC was higher when treated with only the second fraction of the ejaculate than when treated with the first and third fractions [17]. However, studies in pigs suggest that seminal plasma contains factors that may alter spermatozoa, reducing their ability to freeze and fertilise after thawing [18]. Some studies suggest that seminal plasma may affect fertility through changes in the sperm membrane, such as changes in cholesterol levels [19].

Centrifugation is a physical method that removes most seminal plasma, although its effect on sperm quality in different species is controversial. One study reported that the use of centrifugation to remove seminal plasma had no negative effect on sperm quality parameters in dogs [20]. On the other hand, centrifugation is not considered to be completely harmless to the cell and can cause physical damage to cells, removal of capacitation inhibitors and prostaglandins [21].

Centrifugation is a widely used technique to improve sperm quality by selecting sperm with higher motility ratio, reducing abnormalities, and removing non-sperm cells [22]. Moreover, it is a critical step in many assisted reproduction techniques, such as freezing and preparing sperm for shipping in a refrigerated environment. Different centrifugation protocols have been described to improve semen quality using low, medium or high centrifugation intensities [11, 15, 22–26]. The effect of excessive centrifugation force on sperm recovery rates has been investigated, suggesting that high physical pressure against the tube wall may influence the outcome [27–29]. Consequently, some studies have investigated different centrifugation intensities and the addition of different extenders to optimize the process [30–34]. The purpose of this procedure is to remove prostatic fluid and seminal plasma from canine ejaculates in both clinical and experimental settings [35]. Indeed, centrifugation is widely used as the first step of sperm preparation in other species [29, 36–38]. Several studies have shown that equine, bovine and ovine sperm can withstand centrifugation better than human or rodent sperm [30, 39–44]. For example, stallion semen can be centrifuged at 1800–2400 *g* for 5 min without significant adverse effects on semen quality parameters [28, 41, 45, 46]. To note, most semen samples from these species are diluted with an extender prior to centrifugation, a practice not used in standard processing of canine ejaculates [28].

The aim of this study was to evaluate the efficacy and influence of a second centrifugation after 48 h of storage at 5 °C on the sperm quality of canine semen. In clinical practice, shipments of semen material often do not arrive within twenty-four hours, so the authors’ intention was to optimise canine semen storage and handling procedures for practical purposes. In fact, 24 h after cooling, semen parameters are still acceptable for artificial insemination and centrifugation is not considered necessary during this period. After 48 h of cooling, sperm parameters begin to deteriorate, particularly motility, and with this work we have shown how a second centrifugation of the semen restores sperm parameters to the minimum values necessary to ensure an acceptable pregnancy rate. In addition, the correlation between the animal’s body weight and certain sperm parameters after centrifugation was investigated, in line with previous studies showing the variation of some semen parameters at different body weights.

## Methods

### Animals

A total of 45 adult male dogs were enrolled in this study from October 2022 to July 2023 at the Department of Veterinary Sciences of the University of Messina, Italy, for routine evaluation of potential fertility. Informed consent was obtained from the owner of each dog before its inclusion in the study. Inclusion criteria for the animals were based on clinical history, physical examination and reproductive ultrasound of the prostate and testes to exclude pathological conditions, complete blood count, biochemistry, and hormonal profiles. The average age of the animals was 7.3 ± 2.5 years. The subjects were divided into three groups according to their weight. The first group consisted of nine German Dachshunds and six Jack Russell Terriers weighing between 5 and 15 kg (Group 1), with an average age of 7.47 ± 2.26 kg. The second group consisted of ten English Setters and five Pointers, weighing between 15 and 25 kg (Group 2), with an average age of 7.60 ± 2.20 years. The third group consisted of eight Labrador Retrievers and seven German Shepherds, weighing between 25 and 35 kg (Group 3), with an average age of 7 ± 3 kg. Prior to data collection for the study, a detailed general and reproductive history was obtained from each subject.

### Clinical examination and ultrasound findings

A comprehensive physical examination and complete blood analysis were conducted on each subject. In addition, a specific objective examination of the reproductive system was performed, including ultrasonography of male genital tract and semen evaluation. Ultrasound examinations of the testes, epididymis and prostate were performed once on each dog by the same operator using a Mindray M9 ultrasound machine (Mindray, Shenzhen, China) equipped with a linear transducer for the testes and epididymis, instead of micro-convex transducer for the prostate, operating in the frequency range 6.6 to 13.5 MHz. Standardized depth settings were used as much as possible, and overall gain, dynamic range, focal zone, and time-gain compensation were optimized.

### Semen analysis

To minimize defects in the semen stored in the epididymis, such as reduced motility and increased debris [47], a preliminary semen collection was performed 48 h before the examination.

Semen collection was performed in a quiet and suitable environment with a non-slip floor, by manual collection and in the presence of a teaser bitch. The ejaculate was fractionated by discarding the third fraction and immediately examining the first two fractions. The collected semen sample (A) was examined immediately as fresh semen (A_F_). For macroscopic evaluation (volume, colour, odour, and pH), the semen was placed in a falcon tube and kept in a 37 °C water bath. For microscopic evaluation (motility, concentration, morphology and vitality), a 2 µL aliquot of seminal material was placed on a Leja chamber (SC 10-01-04-B, Leja, GN Nieuw-Vennep, NL) and analysed with the aid of a Nikon Eclipse Ni phase contrast optical microscope, equipped with a heated stage, 10x/0.25 Ph1 phase contrast objective, Blaser Scout sca780-54fc digital camera (resolution 782 × 582 pixels; 54 frames per second) and computerized automatic semen analysis system SCA (Sperm Class Analyzer, Microptic Automatic Diagnostic System). The concentration was evaluated either by the SCA system and with a photometer (Accucell IMV Technologies, L’Aigle, France), both of which calibrated for dogs by prior validation with a Makler chamber (Sefi-Medical Instruments, Haifa, Israel). From the results obtained with the spectrophotometric test and the SCA system, the mean concentration was calculated, and this represented the reference value expressed in x10^6^/mL.Motility was analysed by the SCA system, evaluating the following sperm kinematics parameters: total sperm motility (TSM, %), progressive sperm motility (PSM, %), curvilinear velocity (VCL, µm/s), straight line velocity (VSL, µm/s), average path velocity (VAP, µm/s), linearity (LIN, %), straightness (STR, %) wobble (WOB, %).

After staining with eosin/nigrosin, cell morphology was assessed by examining at least 200 spermatozoa per slide in the field at 400x.

Vital-Test kit was used to assess sperm membrane integrity, which provides a green coloration (acridine orange, 1 µL) of nuclei of live spermatozoa with intact plasma membrane and a red coloration (propidium iodide, 1 µL) of dead sperm nuclei, visualized by a fluorescence microscopy (magnification x1000) [48]. For each sample, 200 spermatozoa were examined and the percentage of spermatozoa with an intact membrane was reported.

### Experimental design

At the end of the macroscopic and microscopic evaluation of the sample A_F_ (of each group) was diluted appropriately with CaniPRO™Chill10 extender (Minitüb, Germany) (A_D_). A portion of the diluted semen material was directly chilled by placing the sample at a temperature of 4–6 °C to assess longevity at 24 (T_24_) and 48 (T_48_) hours after chilling.

The remaining part was divided into four 15 mL Falcon tubes, placed in an ALC Pk 121R centrifuge (Thermo, electron corporation, Germany) and centrifuged at 1800 rpm (700x *g*) for 5 min at a temperature of 20 °C to separate the spermatozoa from the rest of the suspension (A_C_). After centrifugation, the spermatozoa formed a pellet at the apex of the tube cone. The supernatant was then aspirated using a vacuum pump (Minitube GmbH & CO, Germany), taking care not to aspirate the pellet, and leaving a minimum of 0.5 mL. After addition of the extender, the pellet was gently resuspended with a micropipette (Pipetman F1000, Gilson, France) until complete dissolution. After resuspension, a 2 µL aliquot of the diluted semen was reassessed under the microscope, while the remainder was stored in the refrigerator for longevity assessment at T_24_ and T_48_ after refrigeration.

The A_C_ sample was submitted to a second centrifugation (A_2C_) after 48 h of storage, following the same procedure, aspirating the supernatant, and resuspending the pellet for a second microscopic evaluation.

**Fig. 1:**
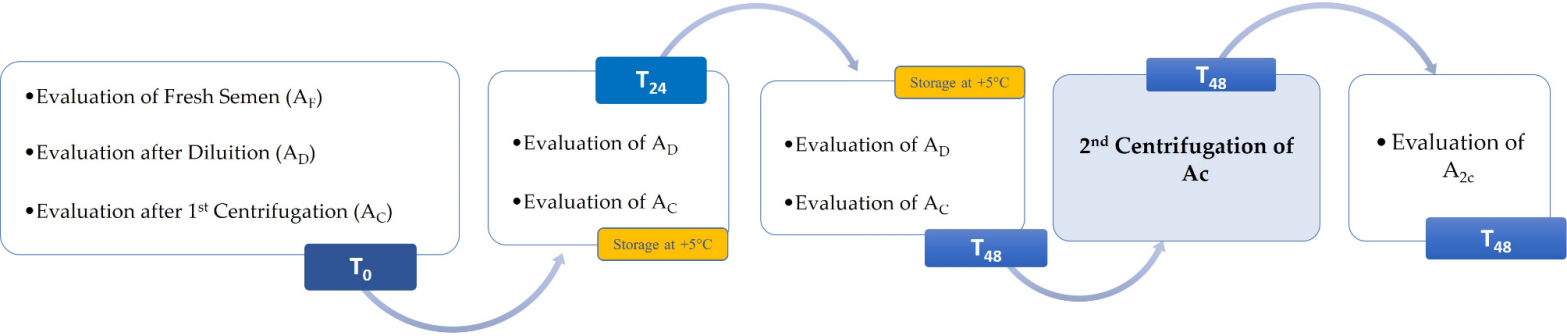
Diagram of treatments and semen evaluations at different timepoints. A_F_ = sample of fresh semen, A_D_ = sample after dilution, A_C_ = sample after centrifugation, A_2C_ = sample after second centrifugation; T_0_ = moment of collection, T_24_ = after 24 h of storage, T_48_ = after 48 h of storage

The collected sperm samples were then analysed at the time of collection (T_0_) as fresh semen (A_F_), after dilution (A_D_) and after centrifugation (A_C_). After 24 (T_24_) and 48 h (T_48_), the refrigerated A_D_ and A_C_ samples were analysed. In addition, at T_48_, the A_C_ semen sample was subjected to a second centrifugation (A_2C_) and then analysed (Fig. 1).

### Statistical analysis

Statistical analysis was performed using GraphPad Prism 7.0 for Windows (GraphPad Software, San Diego, CA, USA). Mann-Whitney U test was used to compare TSM and PSM between: A_F_ and A_D_; A_F_ and A_C_; A_D_ and A_C_ at T_0_; A_F_ and A_D_; A_F_ and A_C_; A_D_ and A_C_ at T_24_; A_F_ and A_D_; A_F_ and A_C_; A_F_ and A_2C_; A_D_ and A_C_; A_D_ and A_2C_; A_C_ and A_2C_ at T_48;_ and to compare morphology and intact plasma membrane among the different groups of weight, among the different typologies of semen, and among different storage temperatures.

Kruskal-Wallis test and T-test were used to compare kinematic parameters between dogs of Group 1, 2 and 3 in A_F_, A_D_ and A_C_ at T_0_, A_D_ and A_C_ at T_24_ and A_D_, A_C_ and A_2C_ at T_48_. Spearman’s Rho test was used to measure the strength of correlation between concentration, total sperm count, TSM, PSM, ejaculate volume and body weight. Differences were considered significant if P values were < 0.05.

## Results

General and reproductive system physical examinations, libido, blood tests, and ultrasound examinations of the 45 dogs showed no alterations. Body weights ranged from 5.5 to 35 kg (median = 24 kg; 25th percentile = 7.75 kg; 75th percentile = 29.5 kg).

Semen parameters including ejaculate volume (mL), sperm concentration (spz x10^6^/mL), TSC (x10^9^), TSM%, PSM%, VCL (µm/s), VSL (µm/s), VAP (µm/s), LIN%, STR%, and WOB%, sperm morphology and membrane integrity at baseline conditions in the three groups of weight are reported in Table 1.

**Table 1:** Semen parameters at baseline conditions in the three groups of weight

Parameter	Mean values ± standard deviation			
	All groups	Group 1 (5–15 kg)	Group 2 (15–25 kg)	Group 3 (> 25 kg)
Ejaculate volume (mL)	6.71±2.76	4.20±0.94	6.27±2.12	9.67±2.44
Concentration (spz x10^6^/mL)	117.91±7.75	110.15±7.53	116.32±9.81	127.29±17.81
TSC (10^6^ spz)	788.80±373.67	457.08±74.93	715.63±201.48	1356.7±258.84
TSM (%)	96.92 ± 2.00	98.56 ± 0.59	97.55 ± 0.60	94.65 ± 1.76
PSM (%)	74.10 ± 6.30	81.33 ± 1.56	69.78 ± 0.60	71.20 ± 6.10
VCL (µm/s)	97.39±18.21	116.74 ± 27.36	94.55 ± 3.09	80.57 ± 23.78
VSL (µm/s)	43.69±3.39	47.60 ± 5.52	46.16 ± 6.57	41.47 ± 3.14
VAP (µm/s)	60.44±9.71	70.09 ± 13.64	57.04 ± 4.82	54.21 ± 8.81
LIN (%)	40.24 ± 1.04	39.04 ± 1.69	40.90 ± 0.93	40.78 ± 0.76
STR (%)	62.81 ± 2.10	63.45 ± 1.19	60.67 ± 3.17	64.31 ± 1.97
WOB (%)	51.76 ± 5.12	55.17 ± 4.82	45.87 ± 8.33	54.24 ± 3.51
Morphology (%)	81.85 ± 4.61	82.23 ± 5.45	82.65 ± 4.27	80.69 ± 4.11
Intact membrane (%)	84.78 ± 2.95	84.91 ± 2.56	86.18 ± 3.07	83.29 ± 2.65

Dogs in the study were split into three groups: Group 1 (5–15 Kg), Group 2 (15–25 Kg) and Group 3 (> 25 Kg). *TSC* total sperm count, *TSM* total sperm motility, *PSM* progressive sperm motility, *VCL* curvilinear velocity, *VSL* straight line velocity, *VAP* average path velocity, *LIN* linearity, *STR* straightness, *WOB* wobble

At T_24_ TSM and PSM in both A_D_ (TSM, *P* < 0.0001; PSM, *P* < 0.0001) and A_C_ (TSM, *P* < 0.0001; PSM, *P* < 0.0001) were lower than in A_F_, however, both TSM and PSM were significantly higher in A_C_ than in the A_D_ (TSM, *P* < 0.0001; PSM, *P* < 0.0001) (Table 2). At T_48_, the TSM and PSM were significantly lower in the A_D_ (TSM, *P* < 0.0001; PSM, *P* < 0.0001), A_C_ (TSM, *P* < 0.0001; PSM, *P* < 0.0001) and A_2C_ (TSM, *P* < 0.0001; PSM, *P* < 0.0001) when compared to the A_F_; however, TSM and PSM were significantly higher in the A_2C_ semen compared to the A_D_ (TSM, *P* < 0.0001; PSM, *P* < 0.0001) and A_C_ (TSM, *P* < 0.0001; PSM, *P* < 0.0001), and in the A_C_ when compared to the A_D_ (TSM, *P* < 0.0001) (Table 2).

**Table 2:** Total sperm motility (TSM) and progressive sperm motility (PSM) at different time points

Parameter	Total cohort Mean values ± Standard deviation							
	T_0_			T_24_		T_48_		
	A_F_	A_D_	A_C_	A_D_	A_C_	A_D_	A_C_	A_2C_
TSM %	96.9 ± 2.0	97.3 ± 1.13	87.3 ± 12.0 ^a^	58.9 ± 13.9 ^c^	73.3 ± 12.8 ^c^	32.0 ± 1.71 ^e^	48.0 ± 8.20 ^d, e^	65.1 ± 11.9 ^d, e,f^
PSM %	71.3 ± 6.31	75.7 ± 6.94	55.0 ± 17.8 ^a^	20.8 ± 5.69 ^c^	39.8 ± 4.36 ^c, d^	3.12 ± 5.31 ^e^	6.18 ± 6.06 ^d, e^	12.3 ± 5.19 ^d, e,f^

^a^= statistically significant lower values than **A**_**F**_ and **A**_**D**_ (*P* < 0.0001)

^b^= statistically significant higher values than **A**_**F**_ (*P* < 0.0001)

^c^= statistically significant higher values than A_F_ (*P* < 0.0001)

^d^= statistically significant higher values than A_D_ (*P* < 0.0001)

^e^= statistically significant lower values than A_F_ (*P* < 0.0001)

^f^= statistically significant higher values than Ac (*P* < 0.0001)

**Fig. 2:**
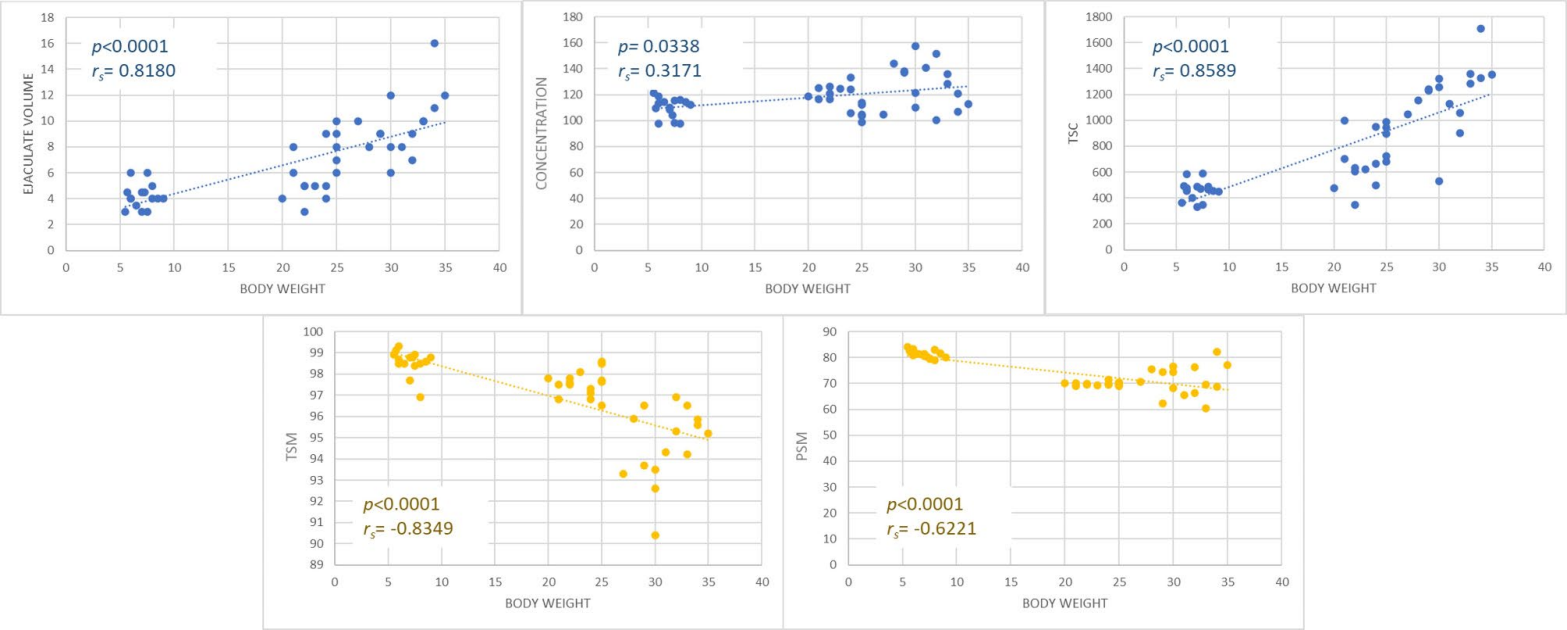
Correlation of body weight with semen factors evaluated. Factors include ejaculate volume, concentration, *TSC* total sperm count, *TSC* total sperm motility, and *PSM* progressive sperm motility

Body weight was positively correlated with ejaculate volume (*P* < 0.0001; r_s_= 0.8), concentration, and, consequently, with total sperm count (*P* < 0.0001, r_s_ = 0.86), while a negative correlation was found between body weight and total sperm motility (*P* < 0.0001, r_s_= -0.83) and progressive sperm motility (*P* < 0.0001; r_s_= -0.62) (Fig. 2).

More specifically, compared to Groups 2 and 3, dogs in Group 1 had higher TSM and PSM in A_C_ at T_24_, higher TSM in A_C_ and A_2C_ semen at T_48_ and higher PSM in A_D_ semen at T_0_. In Group 3, PSM was higher in A_D_ at T_24_ and in A_D_, A_C_ and A_2C_ at T_48_. The results of VCL, VSL and VAP are shown in Table 3.

**Table 3:** Kinematic seminal parameters at different time points in the three groups

Parameter	Group 1 Mean values ± Standard deviation								Group 2 Mean values ± Standard deviation								Group 3 Mean values ± Standard deviation							
	T_0_			T_24_		T_48_			T_0_			T_24_		T_48_			T_0_			T_24_		T_48_		
	A_F_	A_D_	A_C_	A_D_	A_C_	A_D_	A_C_	A_2C_	A_F_	A_D_	A_C_	A_D_	A_C_	A_D_	A_C_	A_2C_	A_F_	A_D_	A_C_	A_D_	A_C_	A_D_	A_C_	A_2C_
TSM %	98.56± 0.59^a^	97.09± 0.53	98.20± 0.53^a^	53.92± 0.56	85.84± 0.53^a^	30.21± 0.53	58.27± 0.60^a^	77.54± 0.91^a^	97.55± 0.60	98.12± 0.60	70.90± 0.59	77.52± 0.55	77.95± 0.56	33.68± 0.78	47.21± 0.73	68.38± 1.19	94.65± 1.76	96.56± 1.42	92.82± 1.33	45.12± 1.41	56.07± 1.29	32.25± 1.34	38.58± 1.33	49.34± 1.08
PSM %	81.33± 1.56^a^	85.45± 1.53^a^	79.94± 1.55^a^	20.55± 4.60	42.68± 1.06^a^	0.91± 0.24	1.85± 0.38	6.72± 0.37	69.78± 0.60	71.40± 0.61	37.83± 0.81	16.68± 0.99	35.14± 0.99	3.25± 0.71	6.21± 0.67	12.43± 0.90	71.20± 6.10	75.91± 6.19	54.20± 4.80	26.81± 4.85^b^	40.73± 5.02	11.53± 4.59^b^	14.67± 4.88^b^	17.52± 4.60^b^
VCL (µm/s)	116.74± 27.36^a^	101.97± 13.59	94.79± 19.71	32.51± 6.91	67.24± 7.15	19.94± 4.08	27.38± 1.20	36.78± 1.8	94.86± 3.57^c^	94.55± 3.09	76.51± 6.14	42.71± 7.53	70.64± 11.95	25.13± 3.25	29.61± 1.95	39.40± 1.91	80.57± 23.78	80.55± 16.69	71.26± 13.55	36.95± 0.62	48.66± 19.11	23.42± 0.83	27.7± 0.74	37.97± 0.11
VSL (µm/s)	47.60± 5.52^a^	61.86± 15.63	46.08± 5.75	12.46± 5.43	28.73± 0.83	5.27± 1.97	9.08± 1.06	16.55± 2.06	42.02± 2.37	46.16± 6.57	45.22± 4.54	20.88± 6.47	36.16± 9.66	8.01± 1.9	10.77± 1.33	18.88± 1.22	41.47± 3.14	44.41± 9.06	34.73± 10.3	15.56± 1.04	23.07± 8.83	6.71± 0.06	9.64± 0.26	18.61± 0.84
VAP (µm/s)	70.09± 13.64^a^	72.75± 15.51	58.05± 9.33	17.94± 5.39	37.81± 0.72	8.89± 2.38	13.97± 0.54	22.22± 1.61	57.04± 4.82	58.35± 4.83	52.79± 1.89	26.52± 6.73	45.04± 10.96	11.98± 1.97	15.21± 1.21	24.31± 1.32^c^	54.21± 8.81	54.26± 10.64	43.51± 11.23	20.82± 1.33	29.02± 11.68	10.85± 0.41	13.87± 0.67	23.57± 0.28
LIN %	39.04± 1.69	58.59± 6.71	49.36± 4.56	36.39± 7.36	42.99± 5.73	25.56± 3.94	33.02± 2.32	44.85± 3.33	40.90± 0.93	48.38± 7.72	59.85± 10.27	47.29± 8.05	50.71± 5.16	31.23± 4.08	36.25± 2.23	47.83± 0.87	40.78± 0.76	54.55± 1.01	48.55± 5.71	41.11± 0.69	47.46± 0.57	28.25± 0.13	34.73± 0.09	48.95± 2.45
STR %	63.45± 1.19	84.21± 4.08	79.49± 2.71	66.65± 8.01	76.07± 3.66	57.66± 5.39	64.76± 4.92	74.21± 3.74	60.67± 3.17	78.46± 4.05	85.44± 5.71	76.84± 6.39	80.06± 1.96	65.71± 5.97	70.58± 3.29	77.54± 0.96	64.31± 1.97	81.31± 0.02	79.29± 2.99	73.46± 1.61	79.87± 1.71	61.07± 0.57	69.41± 1.63	78.82± 2.77
WOB %	55.1± 4.82	70.80± 5.95	61.7± 3.23	54.02± 4.07	56.29± 4.66	44.05± 2.51	51.01± 0.28	60.37± 1.41	45.87± 8.33	61.68± 6.93	69.52± 7.75	61.02± 5.82	63.16± 5.05	47.31± 2.09	51.34± 0.75	61.65± 0.41	54.24± 3.51	67.28± 0.98	60.85± 4.51	55.67± 1.74	59.31± 0.38	46.13± 0.42	50.07± 1.04	62.05± 0.98

^a^= statistically significant higher values in group 1 compared to group 2 and 3

^b^= statistically significant higher values in group 3 compared to group 1 and 2

^c^= statistically significant higher values in group 2 compared to group 3

At T_24_ the percentage was higher in A_C_ than in A_D_ (*P* < 0.001). Finally, at T_48_ normal spermatozoa were higher in A_C_ compared to A_D_ (*P* < 0.001), but higher in A_2C_ compared to A_D_ (*P* < 0.001) and A_C_ (*P* < 0.001). In the statistical comparison of these parameters between A_C_ and A_2C_ at T_48_ among the different groups of weight, only the percentage of intact membrane in the Group 3 was not significantly higher in A_2C_. Results are reported in Table 4.

**Table 4:** Mean percentage of normal sperm morphology and intact plasma membrane after different treatments of semen

Parameter (%)	Mean values ± standard deviation										
	Group 1 (5–15 kg)										
	T_0_				T_24_			T_48_			
	A_F_	A_D_	A_C_		A_D_	A_C_		A_D_	A_C_		A_2C_
Normal morphology	82.23± 5.45 ^a^	78.21± 4.73	84.47± 3.54 ^b^		72.36± 5.60	80.26± 2.87 ^a^		67.96± 5.53	75.37± 2.19 ^a^		79.07± 2.14 ^a, c^
Intact membrane	84.91± 2.56 ^a^	81.35± 3.30	86.51± 1.76 ^b^		77.08± 3.17	81.49± 1.78 ^a^		73.18± 3.28	75.95± 3.12 ^a^		79.01± 2.76 ^a, c^

	Group 2 (15–25 kg)										
	T_0_				T_24_			T_48_			
	A_F_	A_D_	A_C_		A_D_	A_C_		A_D_	A_C_		A_2C_
Normal morphology	82.65± 4.27 ^a^	80.58± 4.14	86.21± 4.06 ^b^		75.33± 3.67	81.07± 3.13 ^a^		69.86± 3.94	75.07± 2.45 ^a^		79.79± 2.48 ^a, c^
Intact membrane	86.18± 3.07 ^a^	83.30± 2.69	88.09± 2.38 ^b^		78.74± 2.57	83.34± 2.18 ^a^		73.98± 2.38	77.80± 2.58 ^a^		79.89± 2.38 ^a, c^

	Group 3 (> 25 kg)										
	T_0_				T_24_			T_48_			
	A_F_	A_D_	A_C_		A_D_	A_C_		A_D_	A_C_		A_2C_
Normal morphology	80.69± 4.11 ^a^	78.38± 4.40	84.03± 4.20 ^b^		74.51± 4.23	79.63± 3.77 ^a^		69.46± 4.18	74.14± 4.28 ^a^		78.48± 3.75 ^a, c^
Intact membrane	83.29± 2.65 ^a^	80.67± 2.68	84.80± 2.58 ^b^		76.64± 3.15	79.82± 2.58 ^a^		71.99± 3.21	74.76± 2.91 ^a^		77.02± 2.94 ^a^

T_0_ = moment of semen collection; T_24_ = after 24 h of refrigeration; T_48_ = after 48 h of refrigeration; A_F_=fresh semen; A_D_=after dilution; A_C_=after centrifugation; A_2C_ = after second centrifugation

^a^= statistically significant higher values than in **A**_**D**_ (*P* < 0.005)

^b^= statistically significant higher values in A_C_ compared to A_F_ and A_D_ (*P* < 0.001)

^c^= statistically significant higher values than Ac (*P* < 0.0001)

## Discussion

Over the past few decades, there has been a significant increase in the demand for canine insemination and, consequently, an increase in the shipment of cooled and frozen canine semen. However, there are biological factors and handling conditions that can affect semen quality during the cooling and shipping process for insemination [49–51]. These factors include standard preparation procedures that can adversely affect sperm viability, motility, and potential fertility. In addition, the presence of prostatic fluid and other plasma components in the semen sample seemed to generate reactive oxygen species (ROS), which are known to be detrimental to spermatozoa [50–53]. However, a recent study has shown that there is no difference in the quality and oxidative stress of sperm cooled and stored at 5 °C for 7 days with or without seminal plasma preservation [54]. Consequently, the improvement of sperm parameters in the cooled seminal fluid after centrifugation was not due to the elimination of seminal plasma, but probably due to a mixture of glucose addition in the extender and the elimination of debris and degradation products to promote reactivation of motility and its maintenance after centrifugation [11].

In the present study, ejaculate volume, concentration, TSC, TSM, PSM, VSL, VCL, VAP, LIN, STR, WOB, percentage of spermatozoa morphology and percentage of intact plasma membrane of spermatozoa at T_0_ were consistent with baseline semen parameters previously reported in the literature [47, 55–59].

The centrifugation of fresh semen seems to reduce sperm quality, as showed by the lower kinematic sperm parameters in A_C_ than A_F_ and A_D_ at T_0_, as already reported by some authors, who quantified the loss of spermatozoa in the supernatant as a function of centrifugation force and time [37]. Similar to our study, the authors added an extender (egg yolk-Tris) before and after centrifugation, before cooling and storage at 5 °C for three days. The sperm samples were centrifuged at 180 x g, 720 x g, 1620 x g, and 2880 x g for 5 min, resulting in sperm losses of 8.9%, 2.3%, 0.4% and 0.06%, respectively [37]. In our study, the percentage of sperm loss after centrifugation at 700 x g was 1.9%, slightly lower than previous results, possibly due to the different extender used and the slightly lower g-force. However, according to another study, the different centrifugation g-forces used did not seem to affect the kinematic parameters when 400, 720–900 g were compared [22]. Moreover, in this study the authors reported a decrease in sperm kinetic parameters (total and progressive motility) after cooling and storage for 24 and 48 h, similar to our results, as well as a decrease in membrane integrity and the percentage of morphologically normal sperm, regardless of the centrifugation technique used [22]. On the other hand, the improvement of sperm quality by centrifugation was evident after cooling and storage of the sperm at 5 °C. Indeed, at T_24_ motility of A_D_ was significantly lower than A_F_, but A_C_ showed better TSM and PMS than A_D_. These results were even more pronounced at T_48_, when a second centrifugation on the semen stored for 48 h (A_2C_) improved sperm kinetic parameters compared to A_D_ and A_C_. All kinematics sperm parameters were significantly higher in A_2C_ sperm compared to A_D_ and A_C_. A significant increase of percentage of morphologically normal spermatozoa and spermatozoa with intact plasma membrane was also observed in A_2C_ compared to A_D_ and A_C_, probably due to the elimination of damaged spermatozoa.

The effects of centrifugation before, after, and before and after cooling was investigated in another study [33]. In contrast to our results, the authors found an improvement in semen that was centrifuged only after cooling compared to centrifugation before cooling and before and after cooling [33]. However, in the aforementioned study, a different extender was used and the second centrifugation on cooled semen was performed after 72 h of storage instead of 48 h, thus results are not perfectly comparable.

In our work, semen differences between breeds belonging to three classes of weight (5–15 kg, 15–25 kg and > 25 kg) have been considered. Indeed, there are numerous dog breeds that vary considerably in size. Several studies have shown an association between canine body weight and total sperm count, but differences in morpho-functional characteristics between breeds and their size remain a topic of debate in the scientific community [56, 60, 61]. It has been shown that there is a significant correlation between body weight and total sperm output (TSO) in dogs [56]. This positive correlation between body weight and TSO suggests that sperm production in dogs is mainly influenced by the total weight of functional testicular tissue, which is higher in larger dogs and lower in smaller dogs [62, 63]. In addition, one study found that dogs with a higher body weight also tended to produce ejaculates with a lower curvilinear velocity, suggesting that their spermatozoa have a lower intrinsic velocity [56]. As suggested in bovine males, increased body weight may interfere with scrotal or testicular thermoregulation, reducing the amount of heat that can be radiated and evaporated from the scrotum. As a result, the temperature of the testes and scrotum may increase, having a negative effect on sperm quality parameters [64, 65]. It is crucial to evaluate not only the different size categories (small, medium and large), but also individual breeds, as differences in semen parameters have been observed [60]. Therefore, further research should focus on specific breeds, taking into account the potential impact of the technical characteristics of computerized semen analysers [66, 67]. With increasing specialization in breed selection and breeding practices, the evaluation of semen handling in dogs becomes even more important to improve reproductive performance.

Some limitations are present in this study. First, no data on potential DNA damage are reported in this paper; second, sperm parameters were evaluated after cooling at T_24_ and T_48_, whereas no information are available on frozen semen and after long-term sperm storage. Thus, future approaches should investigate in detail the effects of a second centrifugation on long-term cryopreserved sperm, considering other sperm characteristics such as sperm membrane integrity, acrosome integrity, DNA fragmentation and kinematic parameters.

## Conclusions

This article contains additional information on semen manipulation techniques. Centrifugation is a quick and easy method to select sperm with higher motility and remove debris. Analysis of the data shows that semen samples that were centrifuged and then cooled at 5 °C had acceptable semen parameters, especially in terms of motility, with a gradual decrease in the serial evaluations after 24 and 48 h, which can be improved by a second centrifugation of the cooled semen after 48 h of storage. This study shows that semen handling has undeniable advantages in terms of preserving the minimum semen characteristics necessary to achieve acceptable pregnancy rates, even beyond 24 h, using cooled semen stored at 5 °C. A limitation of this study may be the lack of data on sperm recovery rate, which may affect the overall understanding of the study results and conclusions. Future studies should evaluate the sperm recovery rate after a second centrifugation of semen refrigerated at 5 °C for 48 h.

## Data Availability

The datasets used during the current study are available from the corresponding author on reasonable request.
